# Short-time cold atmospheric pressure plasma exposure can kill all life stages of the poultry red mite, *Dermanyssus gallinae*, under laboratory conditions

**DOI:** 10.1007/s10493-022-00751-6

**Published:** 2022-10-22

**Authors:** Vanessa Rüster, Henrik Werner, Stephan Wieneke, Georg Avramidis, Lars ten Bosch, Eike Tobias Krause, Christina Strube, Thomas Bartels

**Affiliations:** 1 Institute of Animal Welfare and Animal Husbandry, Friedrich-Loeffler-Institut, Celle, Germany; 2 Institute for Parasitology, Centre for Infection Medicine, University of Veterinary Medicine Hannover, Hannover, Germany; 3 Faculty of Engineering and Health, University of Applied Sciences and Art, Göttingen, Germany; 4 University of Applied Sciences and Art, Hildesheim, Germany

**Keywords:** Poultry red mite, Cold atmospheric pressure plasma, Pest control, Acaricide, Plasma-based pest management

## Abstract

In the present study, the acaricidal effects of cold atmospheric pressure plasma treatment on poultry red mites of different developmental stages have been investigated under laboratory conditions using a dielectric barrier discharge system. A total of 1890 poultry red mites and 90 mite eggs, respectively, were exposed to the plasma under various parameter settings with a single plasma pulse generated using the gas mixture of the ambient air at atmospheric pressure. The results showed that all developmental stages of the poultry red mite could be killed by cold atmospheric pressure plasma treatment. Plasma exposure to mite eggs resulted in a complete 100% hatch inhibition regardless of the parameter settings. Post-exposure mortality rates of larvae, nymphs and adults showed significant differences after utilization of plasma at 10 W for 1.0 s. In addition, the mortality rate increased with progressing time after plasma exposure. An average mortality rate of 99.7% was observed after 12 h in all mites exposed to plasma, regardless of the selected plasma parameter, developmental stage, and nutritional status of the mites. Cold atmospheric pressure plasma has an acaricidal effect on all developmental stages of *Dermanyssus gallinae*, suggesting that it could be developed to an effective method for the control of poultry red mites in laying hen husbandry.

**Supplementary Information:**

The online version contains supplementary material available at 10.1007/s10493-022-00751-6.

## Introduction

The poultry red mite (PRM), *Dermanyssus gallinae*, is one of the most important ectoparasites in laying hen husbandry worldwide. This pest affects more than 80% of laying hen farms in Europe (Sparagano et al. 2009; Flochlay et al. 2017). There are different levels of health and economic impacts associated with PRM infestations. The most direct level of impact is an overall reduced health and well-being of infested animals, e.g., due to skin irritation, restlessness in the barn, weight loss, anaemia, and, in worst cases, sudden death (Mul 2013; Temple et al. 2020; Sparagano et al. 2014) also described PRM infestations to be associated with the development of feather pecking and even cannibalism in laying hens. Furthermore, PRM plays a significant role as a vector for various pathogens such as *Salmonella* species and avian influenza virus (Tomley and Sparagano 2018; Schiavone et al. 2022).

Although the preferred host for PRM are chickens, several other poultry species, pigeons and cage birds as well as wild birds are infested. A cross-infestation of humans and other domestic animals can occur causing, e.g., avian mite dermatitis or gamasoidosis, especially in times of imminent starvation (Rosen et al. 2002; Di Palma et al. 2018; Moroni et al. 2021; Sioutas et al. 2021). Individuals with weakened immune systems are particularly affected by infestations of ectoparasitic hematophagous mites (George et al. 2015). Furthermore, an impaired immune system of infected animals can lead to a higher susceptibility to other diseases (Kowalski and Sokol 2009; Kaoud and El-Dahshan 2010). The third level of impact of a PRM infestation is impaired on poultry husbandries where high degrees of infection are related to a serious economic harm (Flochlay et al. 2017). These can occur in form of production losses, treatment costs subsequent to the infestation, through increased mortality, decreased laying performance, reduced egg weight and quality (Flochlay et al. 2017; Sparagano 2020). PRM control often relies on chemical acaricides (Beugnet et al. 1997). Resistance to acaricides such as pyrethroids, organophosphates, carbamates, and formamidines cause major difficulties in the control of the PRM population (Abbas et al. 2014). Chemical treatment methods are viewed critically, in particular from a consumer protection perspective due to possible chemical residues in eggs and meat. Thus, alternative control methods are needed, especially after the fipronil scandal in Europe in 2017 (Alves et al. 2020), which, among others, result in a higher awareness-level of consumers, concerning applied chemical remedies in the food industry. Besides chemical agents, there are many other different control methods, including biological and physical measures (Maurer et al. 2009; Sparagano et al. 2014; Sparagano 2020). Nevertheless, Flochlay et al. (2017) draw attention to the urgent need for an effective and sustainable method to control PRM.

Following this need for alternative treatment methods we propose the application of cold atmospheric pressure plasma (CAPP). Plasma is an ionized form of gas consisting of free electrons and ions (Fridman 2008). In the physical sciences, plasma is often referred to as the fourth physical state of matter in addition to solid, liquid and gaseous (Fridman et al. 2008). A distinction is made between thermal and non-thermal plasmas. CAPP belong to non-thermal plasmas, where electrons are at high temperature, but the neutral gas and ions are at about room temperature (Fridman et al. 2008). For many applications the temperature properties of thermal plasmas are not required or even undesirable. Non-thermal atmospheric pressure plasmas are used in a wide variety of applications and can be generated in various ways, such as a so-called dielectric barrier discharge (DBD). The DBD is a typical non-equilibrium gas discharge at atmospheric pressure typically generated between two electrodes, of which at least one is covered with a dielectric, restricting the charge transport during the discharge (Xu 2001; Kogelschatz 2003, 2004).

Possible mechanisms of action of CAPP include interactions of different plasma particles like ions and electrons, excited atoms, electric fields, reactive oxygen and nitrogen species, and ultraviolet (UV) radiation with the sample surface (Esrom et al. 1989; Kogelschatz 2003; Laroussi and Leipold 2004; Heinlin et al. 2010; Sutar et al. 2021). CAPP exposure can inactivate a wide range of microorganisms including bacteria, fungi, and viruses (Avramidis et al. 2010; Oehmigen et al. 2010; Sun et al. 2012). In addition, it has numerous applications in medicine such as decontamination of surfaces and treatment of wounds or against cancer cells (Laroussi 1996; Raiser and Zenker 2006; Helmke et al. 2011; Emmert et al. 2013; Metelmann et al. 2016; Boeckmann et al. 2020). However, plasma is not only used in medicine, but also in industrial applications such as chemical and physical modification of surfaces (Rehn and Viöl 2003; Cvelbar et al. 2019; Iqbal et al. 2019). Furthermore, plasma is used in agriculture for the treatment of seeds, decontamination of crops, soil remediation and the control of different pest insects (Zhang et al. 2014; Ohta 2016; Brandenburg et al. 2019; Carpen et al. 2019; ten Bosch 2021; ten Bosch et al. 2022).

The aim of the present study was to determine the acaricidal effects of CAPP on PRM of different developmental stages, and the influence of different plasma variables such as electrical power level and exposure duration on the survival of the mites under laboratory conditions.

## Animals, materials and methods

### Sampling and storage of PRM

Poultry red mites were collected from a litter floor laying hen house at the Institute of Animal Welfare and Animal Husbandry in Celle, Germany. Collection was achieved by using tube traps (length 9.6 cm, diameter 2.6 cm) with rolled-up corrugated cardboard (20.1 × 9.0 cm) according to Safrit and Axtell (1984). The traps were attached under the perches and remained there for 24 h to obtain freshly fed PRM. The experiments started 1 day after sampling. Only vivid and motile PRM were selected for each experiment. The mites were individually placed centrally between the dielectric and the ground electrode (area: 21 × 5 cm) by use of a fine brush. Arrangements to prevent mites from escaping were not necessary because plasma was ignited manually immediately after the mite was set down. Engorged females were placed in Petri dishes (93 × 15 mm), sealed with Parafilm and kept in a climate chamber at 25 °C until egg deposition after 24–48 h. To obtain fasting female adults, individuals were stored at room temperature (20 ± 1 °C) for 7 days until the mites appeared greyish. The differentiation of the developmental stages was carried out with the aid of a stereoscopic microscope (Zeiss Stemi 508; Carl Zeiss, Oberkochen, Germany). Life stages and sex at the adult stage in the live PRM used for the experiment were determined according to Di Palma et al. (2012). To reduce motile activity, the mites were previously cooled to 5 °C in a refrigerator, and the Petri dish was placed on a cooling element tempered to 5 °C during differentiation. For checking purposes, subsamples of the stereo microscopically sorted PRM were embedded in Berlese mixture (Diagonal, Münster, Germany) examined by light microscopy (Olympus CX21; Olympus Europa, Hamburg, Germany).

### Plasma setup

**Fig. 1:**
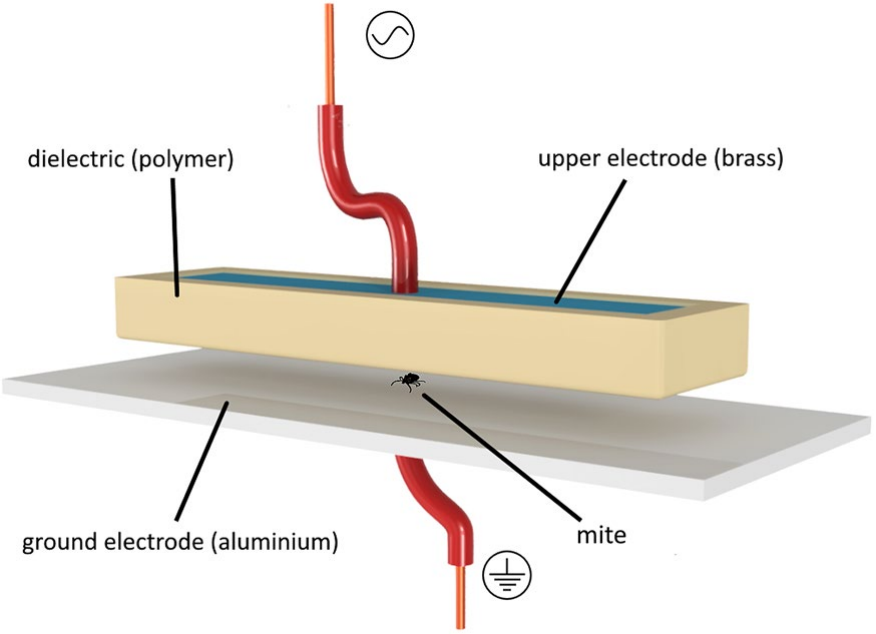
Schematic sketch of the dielectric barrier discharge (DBD) setup

Cold atmospheric pressure plasma (CAPP) was generated by a dielectric barrier discharge (DBD) between two parallel electrodes with the gaseous mixture of the ambient air. The schematic sketch of the used DBD device is shown in Fig. 1. The ground electrode (length: 210 mm; width: 50 mm; height: 3 mm) was made from aluminium and the upper electrode (length: 170 mm; width: 20 mm; height: 1 mm) was made of brass, which was encased in an insulator to provide the plasma from forming an arc. The insulator, also known as dielectric, was made from polylactic acid, a thermoplastic biocompatible aliphatic polyester, using a 3D printer. The dielectric had a thickness of 1 mm and the discharge distance was 1 mm. Two generators—Olympus µs-PNT-2020 (Olympus Europa) and Tantec V-X20 (Tantec, Lunderskov, Denmark)—were used to deliver high voltage from 6 to 9 kV to the electrodes. The Tantec generator produces a sinusoidal voltage, and the Olympus generator operates in a pulsed mode. For all exposure experiments, the applied frequency was 9–17 kHz and the electrical power ranged from 10 to 20 W. A coupled power was used in both generators. The electrode discharge area was 22 cm^2^, resulting in power densities of 0.445 W/cm^2^ for 10 W and 0.91 W/cm^2^ for 20 W by using Tantec HV-X20.

For plasma treatment, mobile PRM individuals or PRM eggs, respectively, were placed individually between the two planar and parallel arranged electrodes. Once the subject was placed, it was immediately exposed to the plasma under various settings using a single plasma pulse. PRM or mite eggs from the control groups were deposited in the CAPP system for the same time as exposed mites without energy input.

### CAPP treatment of PRM eggs

Ovicidal effects of CAPP against PRM eggs were investigated by using different settings of power (10–20 W), frequency (9–13 kHz), voltage (6–9 kV), and exposure time (0.2–1.0 s). Both generators Olympus µsPNT-2020 and Tantec V-X20 were used for this purpose. The experiments with varying voltages and frequencies were performed with the help of an Olympus µs-PNT-2020 generator. In each group, 15 PRM eggs were exposed to CAPP at the following voltage/frequency combinations: 6 kV/9 kHz, 6 kV/13 kHz, 9 kV/9 kHz, 9 kV/13 kHz. Two additional experiments were realized by the Tantec V-X20 generator, which allowed to vary the exposure time. Likewise, 15 PRM eggs each were plasma treated at power levels of 10 W and exposure of 1.0 s as well as 20 W and 0.2 s. For examination of hatchability, a total of 90 plasma-exposed mite eggs and a control group of 10 untreated eggs were placed in Petri dishes. After sealing with Parafilm, the dishes were incubated at a climate chamber at 30 °C and at 70% RH. The egg-hatching rate was determined every 24 h over a period of 5 days.

### CAPP treatment of PRM larvae, nymphal stages and adults

To investigate the effect of CAPP exposure on the mobile PRM developmental stages, the Tantec HV-X20 was applied, as this generator allowed to vary the exposure times of interest for this study. A total of 900 PRM were used, of which 450 mites were treated with CAPP and 450 mites served as untreated controls. To determine the survival rates of the different developmental stages, larvae [n = 30], protonymphs [n = 30], deutonymphs [n = 30], male adults [n = 30] and female adults [n = 30] were each treated with plasma at a power level of 10 W and an exposure time of 1.0 s. Each stage had an untreated control group [n = 30 per run]. The study was performed in triplicate. PRM of the untreated control group and the CAPP-treated experimental group were individually transferred to 2.0 ml cryo-tubes (CryoPure; Sarstedt, Nümbrecht, Germany) using a fine-haired brush. Observations were made in various time units: immediately, 15, 30, 45 and 60 min, and continuously hourly until 12 h after CAPP exposure. The tubes were stored at room temperature (20 ± 1 °C). To testify the death, the mite was touched several times with a fine-haired brush. If there was no reaction or movement, the mite was classified as perished.

### CAPP treatment of engorged and starved female PRM adults

For examination of the influence of the nutritional status, female PRM adults [n = 1440] were exposed to CAPP, consisting of 720 engorged and 720 starved female adults. The engorged [n = 30] and starved [n = 30] female adults were exposed to the plasma for 0.2, 1.0, 1.5 and 2.0 s at power levels of 10 and 20 W, respectively. The untreated control group included engorged [n = 180] and starved [n = 180] female adults. Each replication of the run at 10 W as well as the one at 20 W had a control group with untreated engorged female PRM [n = 30] and untreated starved female PRM [n = 30]. Each experiment was carried out in triplicate. After plasma exposure untreated and CAPP-exposed PRM were placed individually in cryo-tubes as previously described. The survival rate of female adults was recorded directly, after 15, 30, 45 and 60 min, and then hourly after plasma exposure over an observation time of 12 h.

### CAPP treatment of copulating PRM pairs

Furthermore, the effect of cold plasma exposure at 10 W for 1.0 s on copulating mite pairs was also investigated. After exposure, CAPP-exposed mite pairs [n = 13] and an untreated control group of mite pairs [n = 13] were observed for copulating activity, oviposition as well as survival rates every 15 min for 12 h. For this purpose, the pairs were placed in tubes just like the female adults before.

### Statistical analysis

Data on survival were analysed using Kaplan–Meier estimators to obtain survival curves. Survival of PRM was analysed with Gehan–Wilcoxon tests. Further analyses were conducted using a linear model. For all tests, the significance level was determined at α = 0.05. All tests were calculated using RStudio 2021.9.1.372 (RStudio Team 2021) with R v.4.1.2 (R Core Team 2020) and the package *survival* (Therneau 2021). Average survival rates in the text are provided with standard error.

## Results

### Effects of CAPP on hatchability

In the untreated control group, larvae hatched from 100% of the PRM eggs after 5 days. In contrast, no larval hatching was observed in any of the plasma-exposed PRM mite eggs. In the studies with the different combined voltages and frequencies (6–13 kV, 9–13 kHz) a complete hatch inhibition was observed, indicating lethal effects of CAPP to PRM eggs. A complete egg hatch inhibition was equally observed in the treatment with different exposure durations and various power level (10–20 W). During the observation period of 7 days, no further development to the larval stage was detected in the eggs of the plasma-treated groups.

### Effects of CAPP on larvae, nymphal stages and adults

**Fig. 2:**
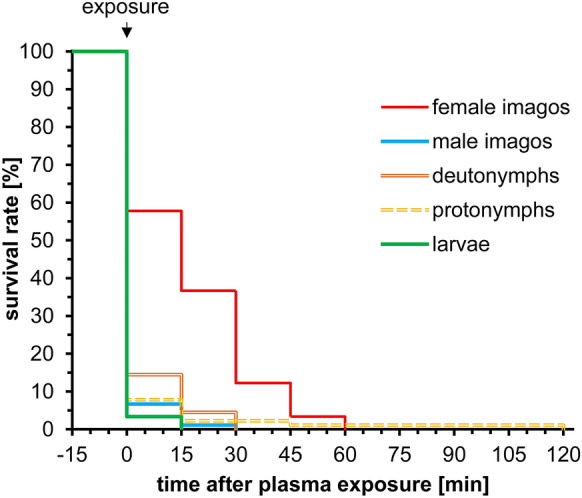
Kaplan–Meier survival curves of different poultry red mite developmental stages after cold atmospheric pressure plasma exposure at 10 W electrical power and an exposure time of 1.0 s

The survival rates of CAPP-exposed larvae, protonymphs and deutonymphs as well as male and female adults are shown in Fig. 2. A CAPP exposure of a power level of 10 W and an exposure time of 1.0 s had a lethal effect on all stages of PRM. However, the experiments showed stage-specific differences in survival rates. Survival rates of larvae, protonymphs, deutonymphs, and male adults decreased significantly (Gehan–Wilcoxon-test: χ^2^ = 131, df = 4, *P* < 0.001) already immediately after CAPP treatment, whereas the survival rate in female adults was still noted at 57.8 ± 1.8% (Fig. 2). The decrease in the survival rates continued with observation time, so that 15 min after CAPP exposure, a 100% mortality was observed in larvae, deutonymphs, and male adults. More time was required to exert the acaricidal plasma effect in protonymphs and female adults but 2 h after plasma exposure a complete cumulative mortality rate of 100% was also achieved in these stages (Fig. 2). Additional data are given in Online Resource 1.

### Effects of CAPP on engorged female adults

**Fig. 3:**
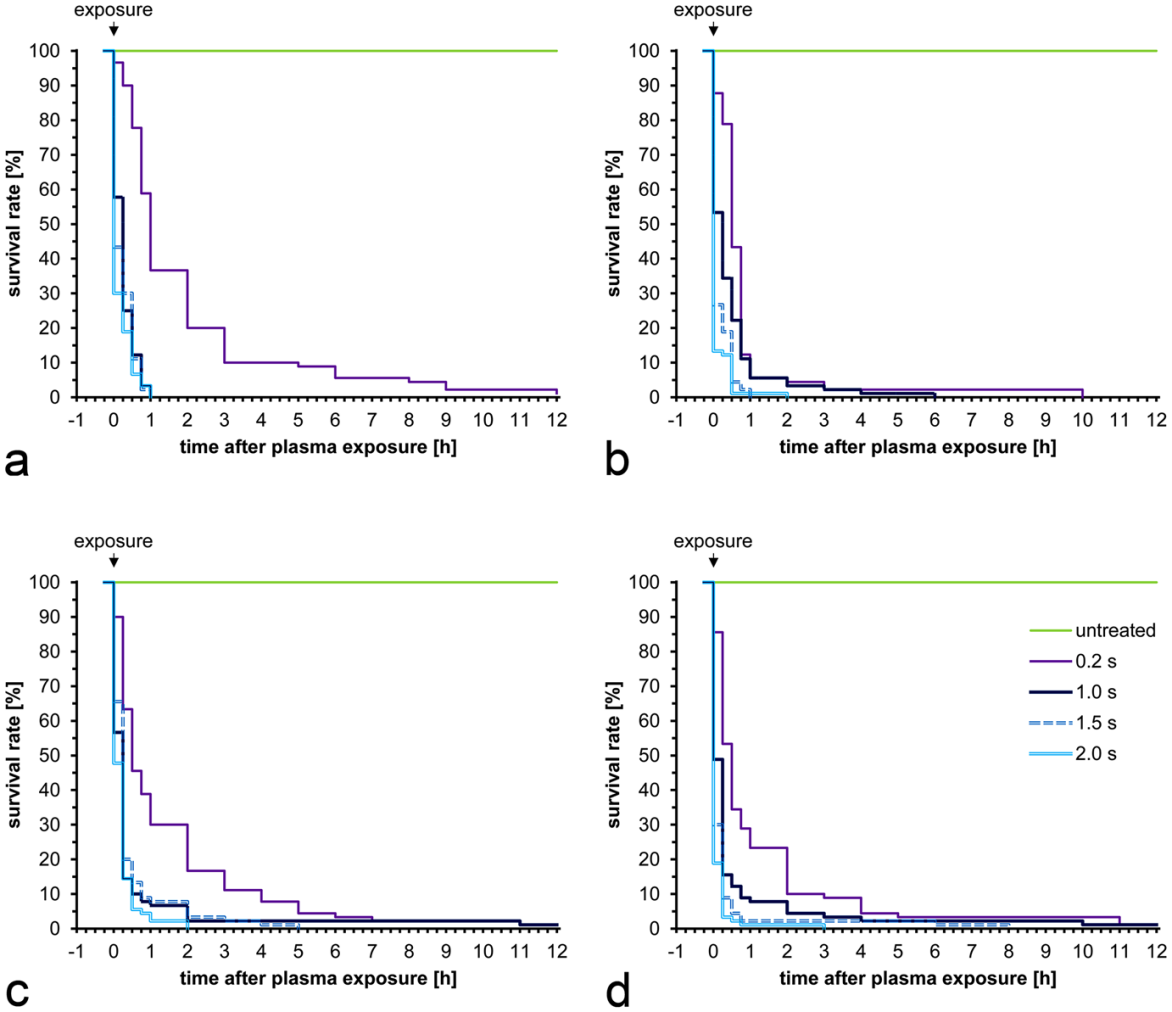
Kaplan–Meier survival curves of female poultry red mite adults of different nutritional conditions after cold atmospheric pressure plasma exposure at various exposure times. **a** Engorged, electrical power 10 W. **b** Engorged, electrical power 20 W. **c** Starved, electrical power 10 W. **d** Starved, electrical power 20 W

The effect of nutritional status on survival after CAPP treatment was studied in female PRM adults. The effect of CAPP on the survival rate of engorged PRM exposed to 10 W of power for exposure times ranging from 0.2 to 2.0 s is shown in Fig. 3a. The lethal effects were evident in the majority of treated PRM immediately after CAPP exposure but also occurred with a time delay (Fig. 3a). Duration of exposure had a significant impact on survival (Gehan–Wilcoxon test: χ^2^ = 343, df = 4, *P* < 0.001). While exposure times of 1.0, 1.5 or 2.0 s led to the death of all PRM within 1 h, the survival rates were significantly higher with exposure times of 0.2 s in comparable time units. Even after an observation period of 12 h, a few individual mites were still alive (Fig. 3a).

Comparable trends in survival rates were observed for exposure times of 1.0, 1.5, and 2.0 s with a CAPP source with an electrical power of 20 W (Fig. 3b). Again, the exposure duration had a significant impact on the survival rate (Gehan–Wilcoxon test: χ^2^ = 321, df = 4, *P* < 0.001). A CAPP exposure of 0.2 s resulted in delayed mortality even at an electrical power of 20 W, but after an observation time of 10 h, all plasma-exposed PRM had died (Fig. 3b). In the control group, no losses were recorded during the observation period (Fig. 3b). Additional data are given in Online Resource 2.

### Effects of CAPP on starved female adults

The plasma exposure of starved PRM had comparable effects on the survival rate as in engorged individuals (Fig. 3a–d). With exposure times of 1.0, 1.5 and 2.0 s under a CAPP source with a power of 10 W, most of the treated PRM died within 1 h (Fig. 3c). A time-delayed decrease in the survival rate could be determined (Fig. 3c). The decrease in survival rates over the observation period of 12 h proved to be statistically significant (Gehan–Wilcoxon test: χ^2^ = 272, df = 4, *P* < 0.001). No animal losses were found in the control groups during the observation period. Comparable influences of the period of time after plasma exposure on the survival rate, which were also statistically verified (Gehan–Wilcoxon test: χ^2^ = 308, df = 4, *P* < 0.001), were also found when using plasma sources with a power of 20 W (Fig. 3d). Control groups showed no losses after the observation period of 12 h (Fig. 3a–d). Additional data are given in Online Resource 3.

The results of our study showed that the survival rate from PRM is significantly influenced by the duration of CAPP exposure (F_4,1784_ = 93,663.57, *P* < 0.0001). Electrical power level (F_1,1784_ = 3.05, *P* = 0.08) and nutritional status (F_1,1784_ = 2.24, *P* = 0.13) had no significant effect on survival rate of female adults. Overall, a mortality rate of 99.7% was observed after the 12 h observation period, irrespective of nutritional status and plasma parameters used such as exposure duration and power.

### Effects of CAPP on copulating pairs

The effect of cold plasma on reproducing paired mites was also investigated. Plasma-treated pairs separated immediately after the plasma pulse and appeared disoriented. Mating activity was not resumed after plasma treatment. After exposure to 10 W electrical power for 1.0 s, 100% of females and 92.3% of males died within 1 h. Complete cumulative mortality was present at the 2-h observation time point. No oviposition was observed in the period between CAPP exposure and death of the mites.

## Discussion

In recent years, studies on application of CAPP on pest arthropods have shown promising results. First studies on plasma-assisted pest control were carried out by Morar et al. (1997), observing that corona discharges at atmospheric pressure were lethal to the hop aphid (*Phorodon humuli*). Mishenko et al. (2000) noticed that a high frequency treatment of combined radiation and plasma discharge resulted in 100% mortality in grain weevils (*Sitophilus granarius*). Keever et al. (2001) used CAPP with helium to treat cigarette beetles (*Lasioderma serricorne*) and observed a mortality rate of 92% in adults after exposure to 70 s irradiation. Non-thermal plasma was also shown to be effective against storage pests like the red flour beetle (*Tribolium castaneum*) and the Indian meal moth (*Plodia interpunctella*) (Abd El-Aziz et al. 2014; Sayed et al. 2021). CAPP was also successfully implemented against human parasites like the head lice (*Pediculus humanus capitis*) (ten Bosch et al. 2019). A comprehensive review of previous studies on cold atmospheric pressure plasma on arthropods over the last 25 years is given by ten Bosch (2021).

To the best of our knowledge, this is the first experimental study describing the acaricidal effects of CAPP on the poultry red mite under laboratory conditions. CAPP exposure of PRM eggs displayed strong ovicidal effects as complete egg hatch inhibition was observed, regardless of the different plasma variables and their combinations used in the study. Importantly, all developmental stages, including mite eggs, can be killed by CAPP treatment. However, the survival rate was significantly influenced by the developmental stage. Especially the larval and both nymphal stages perished promptly after CAPP exposure. The results indicate that female adults appear to be more resistant to CAPP treatment than the other stages. These findings are inconsistent with the results of some research groups that have investigated CAPP on insects. Sayed et al. (2021) observed that the larval and pupal stages of the red flour beetle are more tolerant to direct effects of plasma exposure than the adult stages. Studies on the head lice have shown that adult females are just as susceptible as juvenile stages but adult males showed a lower mortality rate (ten Bosch et al. 2019). In our study, in female PRM adults the lethal effect of CAPP occurred time-delayed, so that complete cumulative mortality was achieved later on. Similarly, it was described by Sayed et al. (2021) that adult red flour beetles are in turn more tolerant to indirect effects such as reactive oxygen species. These could presumably damage the cells, leading to a time-delayed lethal effect of CAPP in insects (Sayed et al. 2021).

The causes of the detrimental effects of CAPP treatment on arthropods are still poorly understood in detail, although first attempts have been made on insects by ten Bosch et al. (2022). Findings such as behavioural changes suggest the involvement of the neuronal and neuromuscular systems as a result of electrostatic surface effects (Donohue et al. 2006). Similarly, Donohue et al. (2008) observed movement disorders in plasma-exposed German cockroaches (*Blattella germanica*). However, in preliminary studies, we could observe that surviving PRM showed disorientation and were motorically limited after plasma treatment. Likewise, several PRM were observed lying on their backs wriggling their limbs until death sets in (Rüster et al. unpubl. obs.). In addition, there are theories that the lethal effects in insects are due to ultraviolet radiation, oxygen species like ozone and nitrogen reactive species (Laroussi and Leipold 2004; Emmert et al. 2013). Abd El-Azis et al. (2014) reported that cold plasma led to oxidative damage in the Indian meal moth caused by generated reactive-oxygen species. Findings from Ziuzina et al. (2021) showed that cold plasma treatment reduces both the respiration rate and the weight in the red flour beetle and affects the levels of oxidative stress markers in adults. However, up to now it is questionable to what extent findings obtained in holometabolous insects, which are significantly larger than PRM and have a heavily sclerotized cuticle, can be readily transferred to PRM.

Our results showed that the survival rates of plasma-exposed PRM depended significantly on the exposure time and, moreover, may be time-delayed. These results correspond to the findings of Donohue et al. (2006) and Sayed et al. (2021) that the mortality rate of plasma-treated insects depends not only on the duration of CAPP exposure but also in the post-plasma exposure period. In our study, PRM demonstrated significant differences in survival rates with respect to the utilized exposure duration. Complete cumulative mortality was achieved more rapidly with prolonged exposure. Furthermore, the data demonstrate that the electrical power level used to generate the plasma did not significantly affect the survival rate. The test groups treated with CAPP at 20 W electrical power did not have significantly higher survival rates than the groups exposed to CAPP at 10 W. Consequently, a lower power is sufficient to achieve the same lethal effect. In addition, the nutritional status in female adults does not appear to have a significant effect on survival time. Both engorged and starved female PRM were susceptible to CAPP exposure.

In recent years the falling efficacy of conventional control methods on PRM infestations have led to increased research into alternative methods (Maurer et al. 2009; Sparagano et al. 2014; Lima-Barbero et al. 2020). However, none of the measures known so far has proven to be sufficiently effective against PRM infestations in the long term (Sleeckx et al. 2019). Integrated pest management (IPM) is currently considered as a strategy that aims at sustainability by combining prevention, monitoring and control measures (Sparagano et al. 2014). The aim of IPM is to reduce the use of pesticides and avoid the development of resistance in order to minimize the risk to humans, animals and the environment. Therefore, the focus of IPM is on the use of non-chemical methods (Decru et al. 2020). Although IPM is currently still predominantly used to control plant pests, potential applications in pest control in livestock are also being explored (Mul 2017). In this context, CAPP could become a useful tool in an IPM strategy to control and manage PRM infestations in layer farms. According to current knowledge, the acaricidal effect of CAPP is based on physicochemical mechanisms, which makes the development of resistance difficult.

CAPP has been proven as highly effective against all developmental stages of PRM, including mite eggs, but data gathering was performed under laboratory conditions, and therefore data have to be considered still as preliminary. Further studies are necessary to examine, whether CAPP also can be used successfully for PRM control in the field in barns occupied by pullets or laying hens, respectively. Since irradiation with cold plasma cannot be realized in the entire building, current development work aims at integrating the plasma sources into devices that are accepted by the nocturnal mites as shelters during the light phase. In this way, sufficiently long dwell times could be achieved, and the mites could be exposed to sufficient plasma in the immediate vicinity of the electrodes. Furthermore, it must be clarified how many systems are necessary in relation to the barn size, the housing system and the number of laying hens in order to have a lasting effect on the mite population. Investigations must clarify also possible influences of barn climate parameters (e.g., dust load, humidity, harmful gases such as ammonia or hydrogen sulphite, etc.) on the functionality of the plasma sources.

## Supplementary Information

Below is the link to the electronic supplementary material.

Supplementary file1 (DOCX 542 kb)

Supplementary file2 (DOCX 545 kb)

Supplementary file3 (DOCX 546 kb)

## Data Availability

The datasets generated during and/or analysed during the current study are available from the corresponding author on reasonable request.
