# Supplementary material for: Short-time cold atmospheric pressure plasma exposure can kill all life stages of the poultry red mite, Dermanyssus gallinae, under laboratory conditions
Source: Exp Appl Acarol. 2022 Oct 22;88(2):139–52. doi: 10.1007/s10493-022-00751-6 (PMC9666290; doi:10.1007/s10493-022-00751-6)
Supplement: Supplementary file 1 — Supplementary file1 (DOCX 542 kb) [file 10493_2022_751_MOESM1_ESM.docx]

Title: Short-time cold atmospheric pressure plasma exposure is highly effective against the poultry red mite *Dermanyssus gallinae*

Journal: Experimental and Applied Acarology

Authors: Vanessa Rüster, Henrik Werner, Stephan Wieneke, Georg Avramidis, Lars ten Bosch, E. Tobias Krause, Christina Strube, Thomas Bartels

Corresponding author: Thomas Bartels, Institute of Animal Welfare and Animal Husbandry, Friedrich-Loeffler-Institut, Celle, Germany. E-mail: thomas.bartels@fli.de

**Table 1** Percental survival rates (mean ± SE) of larvae, nymphal stages and imagos of PRM after CAPP exposure (power level: 10 W; exposure time: 1.0 s) in relation to period after CAPP exposure

|  |  | **Period after treatment** | | | | | |
| --- | --- | --- | --- | --- | --- | --- | --- |
| **Developmental stage** | **[n]** | **directly** | **15 min** | **30 min** | **45 min** | **60 min** | **120 min** |
| larvae | 90 | 3.3±0.0 | no surviving mites detectable | | | | |
| protonymphs | 90 | 7.8±4.0 | 2.2±1.8 | 2.2±1.8 | 1.1±0.9 | 1.1±0.9 | no surviving mites detectable |
| deutonymphs | 90 | 14.4±3.6 | 4.4±0.9 | no surviving mites detectable | | | |
| male imagos | 90 | 6.7±1.6 | 1.1±0.9 | no surviving mites detectable | | | |
| female imagos | 90 | 57.8±1.8 | 36.7±11.9 | 12.2±4.0 | 3.3±1.6 | no surviving mites detectable | |
