# Supplementary material for: Short-time cold atmospheric pressure plasma exposure can kill all life stages of the poultry red mite, Dermanyssus gallinae, under laboratory conditions
Source: Exp Appl Acarol. 2022 Oct 22;88(2):139–52. doi: 10.1007/s10493-022-00751-6 (PMC9666290; doi:10.1007/s10493-022-00751-6)
Supplement: Supplementary file 3 — Supplementary file3 (DOCX 546 kb) [file 10493_2022_751_MOESM3_ESM.docx]

Title: Short-time cold atmospheric pressure plasma exposure is highly effective against the poultry red mite *Dermanyssus gallinae*

Journal: Experimental and Applied Acarology

Authors: Vanessa Rüster, Henrik Werner, Stephan Wieneke, Georg Avramidis, Lars ten Bosch, E. Tobias Krause, Christina Strube, Thomas Bartels

Corresponding author: Thomas Bartels, Institute of Animal Welfare and Animal Husbandry, Friedrich-Loeffler-Institut, Celle, Germany. E-mail: thomas.bartels@fli.de

**Table 3** Percental survival rates (mean ± SE) of starved female imagos after CAPP exposure (power level: 10 W and 20 W) at different exposure times in relation to period after CAPP exposure

| **Power [W]** | **t_exp_** | **[n]** | **Period after treatment** | | | | | | | | | | | | | | | |  |
| --- | --- | --- | --- | --- | --- | --- | --- | --- | --- | --- | --- | --- | --- | --- | --- | --- | --- | --- | --- |
|  |  |  | **Directly** | **15 min** | **30 min** | **45 min** | **1 h** | **2 h** | **3 h** | **4 h** | **5 h** | **6 h** | **7 h** | **8 h** | **9 h** | **10 h** | **11 h** | **12 h** | |
| **10** | control | 90 | 100 | 100 | 100 | 100 | 100 | 100 | 100 | 100 | 100 | 100 | 100 | 100 | 100 | 100 | 100 | 100 | |
|  | 0.2 | 90 | 90.0±1.6 | 63.3±7.2 | 45.6±11.4 | 38.9±11.4 | 30.0±8.3 | 16.7±5.7 | 11.1±3.6 | 7.8±2.4 | 4.4±0.9 | 3.3±1.6 | 2.2±1.8 | 2.2±1.8 | 2.2±1.8 | 2.2±1.8 | 1.1±0.9 | 1.1±0.9 | |
|  | 1.0 | 90 | 56.7±8.3 | 14.4±6.3 | 10.0±5.4 | 7.8±3.6 | 6.7±2.7 | 2.2±0.9 | 2.2±0.9 | 2.2±0.9 | 2.2±0.9 | 2.2±0.9 | 2.2±0.9 | 2.2±0.9 | 2.2±0.9 | 2.2±0.9 | 1.1±0.9 | 1.1±0.9 | |
|  | 1.5 | 90 | 65.6±4.0 | 20.0±9.6 | 13.3±8.2 | 8.9±7.3 | 7.8±6.4 | 3.3±2.7 | 2.2±1.8 | 1.1±0.9 | no surviving mites detectable | | | | | | | | |
|  | 2.0 | 90 | 47.8±8.7 | 14.4±5.1 | 5.6±0.9 | 4.4±0.9 | 2.2±0.9 | no surviving mites detectable | | | | | | | | | | | |
| **20** | control | 90 | 100 | 100 | 100 | 100 | 100 | 100 | 100 | 100 | 100 | 100 | 100 | 100 | 100 | 100 | 100 | 100 | |
|  | 0.2 | 90 | 85.6±2.4 | 53.3±12.9 | 34.4±13.2 | 28.9±15.5 | 23.3±16.4 | 10.0±6.8 | 8.9±5.9 | 4.4±3.6 | 3.3±2.7 | 3.3±2.7 | 3.3±2.7 | 3.3±2.7 | 3.3±2.7 | 3.3±2.7 | 3.3±2.7 | 1.1±0.9 | |
|  | 1.0 | 90 | 48.9±2.4 | 15.6±8.7 | 12.2±10.0 | 8.9±7.3 | 7.8±6.4 | 4.4±3.6 | 3.3±2.7 | 2.2±1.8 | 2.2±1.8 | 2.2±1.8 | 2.2±1.8 | 2.2±1.8 | 2.2±1.8 | 1.1±0.9 | 1.1±0.9 | 1.1±0.9 | |
|  | 1.5 | 90 | 30.0±5.7 | 8.9±7.3 | 4.4±3.6 | 2.2±1.8 | 2.2±1.8 | 2.2±1.8 | 2.2±1.8 | 2.2±1.8 | 2.2±1.8 | 1.1±0.9 | 1.1±0.9 | no surviving mites detectable | | | | | |
|  | 2.0 | 90 | 18.9±0.9 | 3.3±1.6 | 2.2±0.9 | 1.1±0.9 | 1.1±0.9 | 1.1±0.9 | no surviving mites detectable | | | | | | | | | | |

t_exp_: Exposure time
